# Efficacy and safety of endoscopic sleeve gastroplasty versus laparoscopic sleeve gastrectomy in obese subjects with Non-Alcoholic SteatoHepatitis (NASH): study protocol for a randomized controlled trial (TESLA-NASH study)

**DOI:** 10.1186/s13063-021-05695-7

**Published:** 2021-10-30

**Authors:** Lucía Lavín-Alconero, Tatiana Fernández-Lanas, Paula Iruzubieta-Coz, Maria Teresa Arias-Loste, Juan Carlos Rodriguez-Duque, Coral Rivas, Maria Luisa Cagigal, Coral Montalbán, Antonio Lopez Useros, Ana Álvarez-Cancelo, Mar García-Saiz, Javier Crespo-García

**Affiliations:** 1 Marqués de Valdecilla Research Institute (IDIVAL), s/n, Calle Cardenal Herrera Oria, 39011 Santander, Cantabria Spain; 2 Department of Clinical Pharmacology, Marqués de Valdecilla University Hospital, Av. Valdecilla, 25, 39008 Santander, Cantabria Spain; 3 Clinical Trial Agency Valdecilla-IDIVAL, Marqués de Valdecilla University Hospital, Av. Valdecilla, 25, 39008 Santander, Cantabria Spain; 4 Department of Gastroenterology and Hepatology, Marqués de Valdecilla University Hospital, School of Medicine, University of Cantabria, Av. Valdecilla, 25, 39008 Santander, Cantabria Spain; 5 Department of Pathological Anatomy, Marques de Valdecilla University Hospital, Av. Valdecilla 25, 39008 Santander, Cantabria Spain; 6 Department of Endocrinology, Diabetes and Nutricion, Marques de Valdecilla University Hospital, Av. Valdecilla 25, 39008 Santander, Cantabria Spain; 7 Departament of General and Digestive Surgery, Marques de Valdecilla University Hospital, Av. Valdecilla 25, 39008 Santander, Cantabria Spain

**Keywords:** NASH, Obesity, Endoscopic sleeve gastroplasty, Laparoscopic sleeve gastrectomy, Bariatric endoscopy, Bariatric surgery, NAFLD

## Abstract

**Background:**

Non-alcoholic steatohepatitis (NASH) is frequently associated with obesity, and its standard treatment is weight loss with diet and exercise; a dy% weight reduction has been associated with improvement in liver histological and analytical abnormalities. However, less than 25% of subjects achieve this goal. Laparoscopic sleeve gastrectomy (LSG) represents the most common procedure of bariatric surgery, providing effective weight loss and improvement in comorbidities such as NASH, but it is associated with several postoperative complications. Endoscopic bariatric techniques are currently on the rise as a new tool in the fight against obesity, offering patients an alternative to more invasive surgery. However, their efficacy and safety compared with LSG is unclear.

**Methods:**

The TESLA-NASH study is a randomized, controlled, open-label, unicentric clinical trial with a medical device. The aim of this study is to evaluate and compare the efficacy and safety of endoscopic sleeve gastroplasty (ESG) versus laparoscopic sleeve gastrectomy (LSG) in liver histology improvement of patients with obesity +/− metabolic syndrome and NASH. A total of 30 patients will be randomized 1:1 to the experimental or control group.

**Discussion:**

LSG is an effective treatment for weight reduction and for the remission of hepatic alterations. However, LSG is associated with acute and chronic postoperative complications. Bariatric endoscopic techniques promise less invasive and more cost-effective approaches to the treatment of obesity and metabolic comorbidities. ESG represents one of the most promising novel endoscopic interventions and it is mainly proposed for patients with mild-to-moderate obesity, but there are still no guidelines that specify its applicability criteria. This clinical trial will help us apply different tactics to the treatment of obesity and NASH.

**Trial registration:**

ClinicalTrials.gov NCT04060368. Registered on Nov 15, 2019.

## Administrative information

**Table:** 

Title {1}	Efficacy and safety of endoscopic sleeve gastroplasty versus laparoscopic sleeve gastrectomy in obese subjects with NASH: study protocol for a randomized controlled trial (TESLA-NASH study).
Trial registration {2a and 2b}.	Clinical trials NCT04060368
Protocol version {3}	VERSION 2.0 OF 15^TH^ NOVEMBER 2019
Funding {4}	This study is a non-commercial study.
Author details {5a}	Marqués de Valdecilla Research Institute (IDIVAL), Department of Gastroenterology and Hepatology, Deparment Clinical Pharmacology and Clinical Trials agency-IDIVAL. Hospital Universitario Marqués de Valdecilla.
Name and contact information for the trial sponsor	IDIVAL (Instituto de investigación sanitaria Marques de Valdecilla)
Role of sponsor	The authors declare that they have no competing interests. The study was designed and conceived independently from the funding and sponsor institutions, neither of which had a role in the collection, management, analysis or interpretation of data, or the writing of the final manuscript.

## Introduction {6a}

Non-alcoholic fatty liver disease (NAFLD) includes a wide spectrum of hepatic affectation characterized by fat accumulation on the hepatic cells, not attributable to other causes as alcohol or/and certain drugs. Non-alcoholic fatty liver disease (NAFLD) is the most common liver disease worldwide. Its 35 prevalence is estimated between 25 and 40% of adults and its incidence is growing, probably as a 36 result of the increase in obesity and associated metabolic disorders [1]. NAFLD includes a spectrum of 37 pathological situations, ranging from simple steatosis to non-alcoholic steatohepatitis (NASH), and 38 fibrosis and cirrhosis. The later conditions potentially lead to hepatocellular carcinoma (HCC) [2, 3].^.^

At present, NAFLD has turned into the first cause of liver disease in Western countries, with a prevalence of about 30% in the general population [4], due to the current epidemic of obesity and metabolic syndrome. The association is especially strong in morbidly obese patients (BMI greater than 40 kg/m^2^), where more than 90% of present NAFLD, a third of them in the form of NASH, and up to 5–10% of the subjects with progression to cirrhosis [5, 6]. In patients with diabetes mellitus, the prevalence of NAFLD is estimated at 40–70% and NASH at around 22% [7, 8]. In addition, the presence of insulin resistance is related to the severity of liver injury, and the presence of steatohepatitis, cirrhosis, and even the development of complications such as hepatic carcinoma [9]. Dyslipidemia is also frequent in these patients, fundamentally in the form of hypertriglyceridemia and low levels of HDL cholesterol [10, 11]. Overall, 80% of patients present with one of the cardiovascular risk factors that constitute the metabolic syndrome (insulin resistance, obesity, dyslipidemia, and high blood pressure) [12].

The first-line treatment for NASH is the modification of dietary habits and physical activity promoting the loss of weight. A loss of between 3 and 5% of the corporal weight improves steatosis, while to reduce necroinflammatory processes, a weight of at least 10% is needed. The diet must be hypocaloric and individualized [13]. Low-fat diets are recommended in patients with dyslipidemia and low carbohydrate diets for patients with resistance to insulin. Nevertheless, only 25% of subjects reach the target of a loss of weight among 5–10%.

On the other hand, physical activity comprised of aerobic to 1 year [14, 15] improves the hepatic histology of an independent way to the loss ponderal.

Bariatric surgery (BS) is a very effective treatment to induce the loss of weight in subjects with morbid obesity; in fact, surgical procedures are clearly superior to physical exercise and the diet in long-term weight control [16, 17]. Clinical evidences suggest that bariatric surgery in obese patients with NASH can improve all its histological components, including steatosis, inflammation, and fibrosis [18, 19]. Lassailly et al. [18] demonstrated that 1 year after surgery, resolution of NASH occurred in 85% of the patients.

The efficacy of the bariatric surgery on the NASH has been assessed in several previous studies [20–31]; however, these findings have been limited by the large heterogeneity observed in populations and size of the studies. A metaanalysis [32] carried out on 15 studies including 766 patients with hepatic pared biopsies, demonstrated that the steatosis was improved in 91.6% of the patients, the NASH in 81.3% of patients, and the fibrosis in 65.5% of the patients. The global result of these studies also demonstrated that the BS is capable of normalizing the hepatic biomarkers and of improving the histological alterations in patients with NASH, although in some isolated studies there has been observed a minimal progression of fibrosis [29–31].

Nevertheless, in spite of these benefits, a 2010 Cochrane revision suggested that the evidence was insufficient to evaluate the results of the surgery as an option of treatment in patients with NASH [33]. In the same way, a metaanalysis of 2015 suggests that improves the hepatic histology and diminishes hepatic biomarkers, but it also concludes major quality evidences must take place before coming to convincing conclusions on the benefits of the bariatric surgery for the NAFLD [34].

Regarding the safety of BS in patients with NASH, a wide study carried out on the American Nationwide Inpatient Sample demonstrated that the BS collaborates to a global decrease in the mortality of the patients with NASH [35, 36]. But, despite its beneficial effects, complications can arise. Cholelithiasis represents the most common post-surgical complication, which happens in almost one of every three patients bearing cholecystectomies in a higher proportion than the general population which is associated with higher complications after surgeries, prolongation of hospitalizations, and higher mortality rate.

Therefore, although the facts suggest that the bariatric surgery is useful in patients with NASH, this pathology does not constitute at this moment an established indication for the surgery taking into account that there do not exist randomized clinical trials to evaluate the resolution of NASH as a primary objective with this procedure.

At present, the bariatric endoscopy (BE) has emerged as a new therapeutic option for the treatment of obesity in patients who do not have a surgical indication, refuse it or do not comply with conventional treatments. The OverStitch system (Apollo method) demonstrated in 2013 that the use of this technique was sure and feasible in a pilot study with four patients [37]. Later, a study with 20 patients demonstrated a weight loss in 30% of the patients after 6 months [38].

Experience with the BE is, at the moment, scarce. However, the preliminary results are very good [39]. The development of the Apollo method is new, but it has showed adequate without significant adverse events, discharging the first day after the procedure, with good results in multicenter studies and showing significant weight loss during the first 2 years. Most of the weight loss takes place during the first 6 months after the procedure, and the weight loss is maintained or increased at months 12 and 24 [40]. Even though BE works well across the spectrum of obesity, including subjects with BMI over 40, the duration of the long-term effect is still unknown. Patients who undergo this procedure achieve significant improvement in the high blood pressure (HBP), cholesterol, and HbA1C levels control as well as improved liver function tests and an improvement in their obstructive sleep apnea disorder [40]

### Objectives {7}

#### Hypotheses

In patients with non-alcoholic steatohepatitis (NASH) and obesity, ESG will achieve weight loss and improvement in metabolic comorbidities and liver histology equal to or greater than LSD with a lower rate of adverse effects.

#### Objective

The objective is to evaluate the efficacy of ESG + lifestyle modification versus LSG + lifestyle modification in resolving NASH without worsening of fibrosis.

#### Trial design {8}

The TESLA-NASH study is a randomized, controlled, open-label, unicentric clinical trial with a sanitary product, designed to evaluate the efficacy of two gastric techniques (endoscopic versus laparoscopic) in the resolution of NASH without worsening liver fibrosis.

- Experimental group: ESG + lifestyle modifications
- Control group: LSG + lifestyle modifications

A total of 30 eligible participants will be randomly assigned (stratified by the presence or absence of type II diabetes mellitus) to the experimental group or the control group with a 1:1 ratio. The participants will be recruited at the Hospital Marqués de Valdecilla from June 2020 to June 2021.

## Methods: participants, interventions, and outcomes

### Study setting {9}

The study will be performed in the Hospital Marqués de Valdecilla, a third-level hospital located in the north of Spain. The Clinical Trials agency and SCReN were responsible for obtaining authorization from the local ethics committee (EC) and notifying the Spanish Agency of Medicines and Medical Devices of the start-up of the study. The study was approved by the Ethical Committee of Cantabria.

### Eligibility criteria {10}

The patient selection will be carried out by the gastroenterologist at daily check-ups. The informed consent will be signed by patients before their inclusion in the study. Patients must present obesity associated or not to a metabolic syndrome according to the criteria of NCEP ATP III, and a NASH diagnosis according to the histological parameters.

#### Inclusion criteria

1. Subjects between the ages of 18 and 60 (inclusive) at the time of signing the informed consent form.
2. They must provide a signed written informed consent and agree to comply with the study protocol.
3. BMI ≥35–45 kg/m^2^ and ≤30–35 kg/m^2^ associated or not with metabolic risk factors (diabetes mellitus, hypertension, dyslipidemia), and subjects with a BMI ≥30–34.9 and ≤45 kg/m^2^ if type 2 diabetes mellitus is associated.
4. Histological confirmation of steatohepatitis in a diagnostic liver biopsy (biopsy obtained in 6 months prior to randomization or during the screening period) with the following:
  - NAS score ≥ 4 with ≥1 point for each component.
  - Degree of fibrosis < 4 according to the NASH CRN staging system.
5. For patients with fibrosis ≤ 1, at least one of the following characteristics must be associated:
  - Metabolic syndrome (NCEP ATP III definition)
  - Type II diabetes mellitus.
  - HOMA-IR > 6
6. Absence of other causes of chronic liver disease (alcoholic, viral, autoimmune, cholestatic disease, Wilson’s disease, α1AT deficit, hemochromatosis)
7. Subject must agree to undergo a liver biopsy 96 weeks after the endoscopic or surgical procedure.

#### Exclusion criteria

1. Known heart failure (grades I–IV of the New York Heart Association classification)
2. History of effective bariatric surgery in the 10 years prior to selection.
3. Patients with a history of clinically significant cardiovascular events in the 6 months prior to selection such as an acute cardiovascular event, stroke, transient ischemic attack, or coronary heart disease (angina, myocardial infarction, revascularization procedures).
4. A weight loss of more than 5% in the 6 months prior to randomization.
5. Current or history of significant alcohol consumption (< 5 years). For men, significant consumption is usually defined as more than 30 g of alcohol per day. For women, defined as more than 20 g of alcohol per day.
6. Liver cirrhosis.
7. Non-cirrhotic portal hypertension.
8. Presence of esophageal and/or gastric varices.
9. Hepatocarcinoma.
10. Portal thrombosis.
11. Pregnancy.
12. Any other medical condition that may diminish life expectancy to <2 years, including known cancers.
13. Indications of any other untreated, unstable or clinically significant immunological, endocrine, hematological, gastrointestinal, neurological, neoplastic, or psychiatric disease.
14. Mental instability or incompetence such that the validity of the informed consent or the ability to comply with the study is uncertain.
15. Positive antibodies to human immunodeficiency virus.
16. Decompensated liver disease measured as follows:
  - Aspartate aminotransferase (AST) and/or ALT > 10 x upper limit of normality (LSN)
  - Total bilirubin > 25 μmol/l (1.5 mg/dl).
  - International normalized ratio > 1.4.
  - Platelet count < 100 000/mm^3^.
17. Serum creatinine levels > 135 μmol/l (> 1.53 mg/dl) in men and > 110 μmol/l (> 1.24 mg/dl) in women.
18. Significant renal disease, including nephrotic syndrome, chronic renal disease (patients with markers of liver injury or estimated glomerular filtration rate [eGFR] of less than 60 ml/min/1.73 m^2^). If an abnormal value is obtained at the first screening visit, eGFR measurement may be repeated before randomization within the following time frame: minimum 4 weeks after the initial test and maximum 2 weeks before the intended randomization. Repeated abnormal eGFR (less than 60 ml/min/1,73 m^2^) leads to exclusion from the study.

### Who will take informed consent? {26a}

The informed consent of the TESLA-NASH study will be signed by all the subjects before their inclusion in the study. Additionally, by daily clinical practice, patients randomized in the experimental group will sign the endoscopic gastroplasty-specific informed consent form and patients in the control group will sign the laparoscopic vertical gastrectomy informed consent form.

### Additional consent provisions for collection and use of participant data and biological specimens {26b}

No collection samples

### Interventions

#### Explanation for the choice of comparators {6b}

Patients with obesity with or without metabolic syndrome, according to NCEP ATP-III (National Cholesterol Education Program’s Adult Treatment Panel III Report), and NASH diagnosed by liver biopsy will be included in the study.

After 10 weeks of the screening period, the patient will be randomized in experimental o control intervention.

#### Intervention description

The interventions and comparators description is shown in Table 1.

**Table 1:** Interventions and comparators description

Groups	Interventions
**Experimental group**	The endoscopic technique is defined as a gastric restriction by means of continuous sutures of the entire gastric wall of the antrum and body, transmurally, in order to simulate a gastric sleeve, in the same way as the sleeve gastrectomy surgery. Gastroplasty is performed using an endoscopic suture system (Overstitch, Apollo endosurgery Inc., Austin, Texas, USA) inserted into a dual-channel endoscope (GIF-2T160.Olympus Medical System Corp., Tokyo, Japan)
**Control group**	The minimally invasive surgical technique is defined as a gastric restriction by means of excision of approximately 80% of the stomach along the greater curvature.

The surgery, LSG, will be performed by the study surgeons while the ESG will be carried out by the endoscopists

#### Criteria for discontinuing or modifying allocated interventions {11b}

In accordance with the revision of the Declaration of Helsinki and current regulations, the patient has the right to withdraw from the study at any time and for any reason, without prejudice to future medical care by his doctor and/or a referral site.

Patients who have withdrawn from the study cannot be included again in the study.

A patient may leave the study for any of the following reasons:

- Withdrawal of the informed consent and/or refusal of treatment and/or lack of cooperation.
- Death of the patient.
- Decision of the principal investigator and/or sub-investigator in charge, if it is in the patient’s interest to stop the study.

#### Strategies to improve adherence to interventions {11c}

To verify the effectiveness of each technique and ensure patient’s safety, follow-up visits will occur after the interventions. Seven follow-up visits will be carried out over 24 months where the variables described in the protocol will be assessed by means of biochemical cholesterol, biomarkers of inflammation and lipid tests changes, cardiovascular events, quality of life and physical activity questionnaires progress, liver biopsy, abdominal ultrasound and non-invasive assessments of fibrosis, post-intervention dietary and lifestyle recommendations and revisions, records of the concomitant medication during the follow-up, and an evaluation of the safety by controlling adverse events and adverse reactions.

#### Relevant concomitant care permitted or prohibited during the trial {11d}

Any concomitant medication is allowed within daily clinical practice. The concomitant medication administered will be registered in the clinical report form (CRF) of the clinical trial.

#### Provisions for post-trial care {30}

This is a non-commercial study carried out by Spanish researchers whom are employees of the national public health system. The sponsor of the study is a non-profit organization, IDIVAL health research institute.

There is no compensation for the subjects participating in the clinical trial.

### Outcomes {12}

#### Primary outcome

Assessment of the efficacy of endoscopic vertical gastroplasty + lifestyle modification against laparoscopic vertical gastrectomy + lifestyle modification in the histological improvement of NASH from baseline to week 96 by the following:

- Improvement of fibrosis in at least 1 stage without worsening of NASH.
- Resolution of NASH without worsening of fibrosis.

The resolution of NASH is defined as the histopathological disappearance of ballooning and the disappearance or persistence of minimal lobular inflammation (grade 0 or 1) using the NASH CRN scoring system with a type of general lesion that does not qualify as steatohepatitis. Worsening of fibrosis is defined as the progression of at least one stage.

For all the patients, a basal liver biopsy should be obtained prior to the execution of the therapeutic technique and a subsequent biopsy 96 weeks after the procedure should also be obtained.

To avoid inter-observer variability, the histological evaluation will be assessed by two local pathologists independently.

#### Secondary outcomes

Evaluation of the following histological changes after 96 weeks of treatment according to NASH-CRN criteria:

- Improvement of at least 1 point in the different components of the NASH-CRN score (steatosis, liver ballooning, and lobular inflammation)
- Resolution of fibrosis
- Fibrosis improvement along with NASH improvement, defined as at least 1 stage improvement in fibrosis and at least 2 points less in NAFLD activity score (NAS) with at least 1 point of improvement in ballooning and lobular inflammation.
- Improvement in NASH score of at least 2 points with no worsening of fibrosis.

Evaluation of potential complications and morbimortality related to the procedures such as bleeding, bronchial aspiration, infections, suture dehiscence, and death.

Evaluation of the following secondary endpoints after 96 weeks of treatment:

- Cardiovascular events: Ischemic heart disease, stroke, peripheral arterial disease, heart failure, and cardiomyopathy.
- Liver-related death.
- Liver profile changes and liver function biomarkers.
- Changes in non-invasive markers of fibrosis and steatosis.
  - Serological markers: NAFLD fibrosis score, FIB-4, and Hepamet score. Several predictive models of advanced fibrosis are based on formulas using clinical and analytical parameters.
  - FibroScan and CAP: Transitional elastography or FibroScan is the most widespread technique in the non-invasive diagnosis of liver fibrosis. It measures by ultrasound the speed of propagation of low-frequency waves through the liver parenchyma. CAP is an application of FibroScan that measures the degree of attenuation of the ultrasound wave transmitted through the liver proportional to the amount of liver fat.
  - DEMILI: Computerized optical analysis software of conventional liver magnetic resonance images that determines a number of optical biomarkers that allow the detection of NASH and the prediction of significant fibrosis.
  - Metabolomic profile: Owl-liver. Serum analysis for the determination of fatty liver disease and NASH diagnosis based on the determination of more than 500 serum metabolites by liquid chromatography coupled to mass spectrometry. Using the method developed by the company OWL (ONE WAY LIVER, S.L., Derio -Vizcaya-).
- Changes in lipid parameters: Cholesterol, LDL, HDL, and triglycerides
- Variation in body weight.
- Changes in endothelial dysfunction biomarkers: ICAM-1, VCAM-1, E-selectin, P-selectin, angiopoietin-2, endocan, RBP4, OPG, and ADMA.
- Changes in macrophage activation biomarkers: Gal 3, Gal-3BP, sCD163, and sCD14.
- Changes in the glucose homeostasis biomarkers and insulin resistance: glucose, hemoglobin A1c (HbA1c), homeostatic model assessment for insulin resistance (HOMA-IR), adiponectin, retinol-binding protein 4 (RBP-4), monocyte chemoattractant protein-1 (MCP-1), plasminogen activator inhibitor-1 (PAI-1), leptin, resistin, visfatin, and apelin.
- Changes in the intestinal microbiota by sequencing the 16S rRNA in stool samples.
- Changes in inflammation biomarkers: fibrinogen, hs-CRP, haptoglobin, TNF-alpha, IL-6, and IL-1β.
- Changes in serum expression of incretins: GLP-1, GIP, and YY peptide.
- Changes in the cardiovascular risk profile.
  - Study of classic risk factors: Framingham scale.
  - Study of emerging vascular risk factors: hcPCR, homocysteine, and Lp(a)
  - Lipid biomarkers: PON1 and PCSK9
  - Qualitative alterations of lipoproteins.
  - Evaluation of endothelial function by digital pulse plethysmography (ENDO-PAT2000 -Itamar Medical Inc., Caesarea, Israel-).
  - Evaluation of the presence of subclinical atherosclerosis by carotid ultrasound.
- Changes in mineral metabolism parameters: Parathormone (PTH), 25-hydroxy-vitamin D (25OHD), N-terminal propeptide of collagen type I (PINP), and beta-cross laps (β-CTX).
- Changes in serum levels of gastrointestinal hormones: glucagon-like peptide-1 (GLP-1), glucose-dependent insulinotropic polypeptide (GIP), tyrosine thyrosine peptide (PYY), ghrelin, oxymodulin (OXM), cholecystokinin (CKK), and fibroblast growth factor 19 (FGF-19).
- Changes in the quality of life (36-item shortened health questionnaire [SF-36]), eating habits (Eating Habits Questionnaire), and physical activity (Global Physical Activity Questionnaire [GPAQ]) based on patients reported outcomes
- Safety and tolerability: number of adverse effects resulting from treatment during the study.
- Economic evaluation of both interventions with a cost-effectiveness analysis.

#### Participant timeline {13}

##### Participant timeline

**Table:** 

PROCEDURES	WEEK 2	WEEK 8	WEEK 12	WEEK 24	WEEK 48	WEEK 72	WEEK 96
**Liver biopsy**							**X**
**QL questionnaires**					**X**		**X**
**Cardiovascular events**							**X**
**Fecal samples**							**X**
**Blood analysis**		**X**	**X**	**X**	**X**	**X**	**X**
**Safety: SAE and RAE**	**X**	**X**	**X**	**X**	**X**	**X**	**X**
**Dietary recommendations**	**X**	**X**	**X**	**X**	**X**	**X**	**X**
**Fibrosis evaluation**							**X**
**Ultrasound proof**					**X**		**X**

##### Sample size {14}

Previous studies have shown a resolution of NASH in the 85% of patients undergoing a bariatric surgery (69.6% in gastric tubing surgery) and a resolution of 25% in patients with a lifestyle intervention. A bilateral ratio comparison test was used for sample size calculation with a confidence level of 99%, a statistical power of 90%, and an assumption of 7% loss. Obtained sample to observe differences is *n*=30 (15 per treatment arm).

##### Recruitment {15}

Potential candidates will be reviewed in daily consultation by the gastroenterologist and endocrinologist. Patients with obesity, with or without metabolic syndrome and a NASH diagnosed by liver biopsy, will be evaluated.

### Assignment of interventions: allocation

#### Sequence generation {16a}

Randomization will be carried out on all eligible subjects using a computer system. At the randomization visit, eligible subjects will be randomized 1:1 for the following groups:

- Experimental group: ESG + lifestyle modifications
- Control group: LSG + lifestyle modifications

Randomization will be stratified into 2 strata defined by diabetes status

#### Concealment mechanism {16b}

Once the patient is selected for inclusion in the study by the site’s investigator and eligibility has been evidenced, the study coordinator will proceed with the randomization of the patient by writing an email to the following email address: lrasines@idival.org. The person in charge of the list will reveal the patient’s assigned code, taking into account the stratification in terms of the presence or absence of diabetes type 2, along with the assignment to the experimental or control intervention. The person in charge of this list is an employee of the same institution but not involved in the selection of patients.

#### Implementation {16c}

Non-recruitment involved staff will inform the study coordinator of the randomization number and treatment group assigned. Then, the patient is notified of the date of the intervention.

#### Assignment of interventions: blinding

Given the nature of this study where surgical procedures are performed versus an endoscopic procedure, the study staff cannot be blinded.

#### Who will be blinded {17a}

Not applicable

#### Procedure for unblinding if needed {17b}

Not applicable

#### Data collection and management

##### Plans for assessment and collection of outcomes {18a}

The coordination of the study is carried out by the Clinical Trials agency, on behalf of the study sponsor (IDIVAL). The review of the data is carried out by the Clinical Research Associate (CRA) from SCReN (Spanish Clinical Research Network) according to the monitoring plan to ensure the validity of the results obtained.

Deviations detected during study monitoring will be reported to the regulatory authorities according to their severity, classified as mild or serious.

Serious deviations are defined as procedures that affect the safety, rights, and welfare of the patients included in the study as well as the quality and integrity of the data obtained, while minor deviations are defined as procedures that do not affect the safety, rights, and welfare of the patients or the quality of the data obtained.

##### Plans to promote participant retention and complete follow-up {18b}

All patients included in the study will be followed for 96 weeks after the intervention (control vs. experimental) to guarantee the patient’s safety, and all the obtained data will be recorded.

Patients who withdrawn from the study because of the criteria mentioned in section 11b will be followed according to the daily clinical practice of the site.

##### Data management {19}

Data collection will be carried out by the study coordinator and the data obtained will be monitored by the CRA. Study-generated data will be collected in a paper case report form (CRF) ad hoc.

##### Confidentiality {27}

In order to avoid any association of personal data with the data obtained, patients will be anonymized by alphanumeric code (Site (01)-01). The research team will have access to the data of patients included in the study, and a list with patients’ identification will be done and keep on the investigator’s file. All the generated data will be recorded in the CRF in an anonymized form.

Personal data will be processed in accordance with Regulation (EU) 2016/679 of the European Parliament and of the Council of 27 April 2016 on the protection of individuals with regard to the processing of personal data and on the free movement of such data, and the relevant local laws.

The data collected for the study shall be identified by an alphanumeric code (Site (01)-01) in such a way that it is not possible to identify the patient. Only the investigator and authorized persons involved in the study will have access to this code and undertake to use this information exclusively for the purposes of the study. All the generated data will be recorded in the CRF in an anonymized form.

Members of the Clinical Research Ethics Committee or Health Authorities may have access to this information in compliance with legal requirements. The confidentiality of this data will be preserved, and it cannot be linked to personal data, even if the results of the study are published.

##### Plans for collection, laboratory evaluation, and storage of biological specimens for genetic or molecular analysis in this trial/future use {33}

Not applicable

### Statistical methods

#### Statistical methods for primary and secondary outcomes {20a}

Data will be analyzed using SPSS version 19. A descriptive baseline and end-of-follow-up analysis (week 96) of the data will be performed using the mean and standard deviation or the median and range where appropriate for quantitative data and percentage for qualitative data. Quantitative variables will be compared with Student’s *t* test and qualitative variables will be compared with *X*^2^. When variables do not follow a normal distribution, non-parametric tests should be used. Paired data tests will be used to analyze baseline and 96-week results.

All changes in efficacy parameters endpoints will be analyzed in an intention-to-treat and per-protocol analysis and grouped by randomized treatment.

The Intent-to-Treat (ITT) population will include all subjects randomized to treatment. The per-protocol (PP) population will be a subset of the ITT population, excluding subjects that violate key protocol criteria, with the exact list of exclusion reasons to be included in the statistical analysis plan (SAP).

The primary efficacy endpoint, by histopathological improvement of fibrosis by at least 1 stage without any worsening of NASH, and histopathological resolution of NASH without any worsening of the stage of fibrosis will be conducted on the intention-to-treat (ITT) population; so all randomized subjects will be included in the primary analysis.

Secondary endpoints analyzed by descriptive statistics and inferential statistics will include ANOVA. Non-parametric tests may be utilized such as Wilcoxon rank-sum.

For all tests, we will use 2-sided *p* values with alpha < 0.05 level of significance.

All safety variables (i.e., adverse events, laboratory data, vital signs, and ECG) will be summarized by treatment for all patients of the safety set. Descriptive statistics using appropriate summary statistics will be calculated for quantitative safety data as well as for the difference to baseline, when appropriate. In addition, a shift table describing out-of-normal range shifts will be provided for clinical laboratory results.

#### Interim analyses {21b}

Not applicable

#### Methods for additional analyses (e.g., subgroup analyses) {20b}

Not applicable

#### Methods in analysis to handle protocol non-adherence and any statistical methods to handle missing data {20c}

Not applicable

#### Plans to give access to the full protocol, participant-level data, and statistical code {31c}

Not applicable

### Oversight and monitoring

#### Composition of the coordinating center and trial steering committee {5d}

Not applicable

#### Composition of the data monitoring committee, its role, and reporting structure {21a}

The development of this clinical trial with a sample size of 30 patients will help us to know the first results regarding the applied techniques. There are no plans to hold a monitoring data committee because it is assumed that these are safe techniques already developed in normal practice.

#### Adverse event reporting and harms {22}

The investigators will be responsible for collecting all the adverse events in the clinical history based on those referred by the patient spontaneously or by an interview in the follow-up visits. The causality of the adverse event with the intervention will be evaluated and recorded in the clinical history and in the CRF.

#### Frequency and plans for auditing trial conduct {23}

The CRA will verify 100% of the events and adverse reactions that occur in all the follow-up visits and the rest of the variables described in the protocol. A monitoring plan is established for the follow-up of the clinical trial.

#### Plans for communicating important protocol amendments to relevant parties (e.g., trial participants, ethical committees) {25}

All amendments to the protocol or informed consent will be submitted to the Ethics Committee- Cantabria to get subsequent approval. These amendments will be updated in the clinical trials registry at ClinicalTrials.gov.

#### Dissemination plans

The results obtained will be published in journals of impact and in congresses related to the subject matter of the protocol.

## Discussion

NASH is a prevalent and worrying disease, given the increased cardiovascular risk that patients with NASH possess. Therefore, lifestyle modification is the first line of treatment, which includes diet modification, sustained weight loss, and increase in exercise.

Gastric bypass surgery and restrictive surgical treatments have been successful in improving metabolic syndrome, insulin resistance, and liver histology in obese patients. Currently, endoscopic bariatric techniques which are less invasive are being developed, showing fewer complications, lower sanitary cost, and gastric restrictions with similar characteristics to the traditional surgical methods. The endoscopic sleeve or vertical gastroplasty (ESG) with the OverStitch system (Apollo Endosurgery, Austin, TX, USA) is one of the most promising ones. However, currently, any surgical or endoscopic technique is accepted as a standard treatment for NASH.

This is the first randomized clinical trial in NASH patients with obesity associated or not with a metabolic syndrome that will assess a bariatric endoscopic procedure in comparison with bariatric surgery. The study will evaluate liver histology improvement in each of the fundamental histological aspects of non-alcoholic fatty liver disease: steatosis, inflammation, and fibrosis. In addition, the change in the cardiovascular risk of treated patients and numerous disease-related biomarkers will be analyzed.

### Trial status

The current version of the protocol is 2.0 on November 15, 2019.

The first patient inclusion for the study occurred in June 2020.

The recruitment period is 1 year (June 2021).

## Data Availability

Not applicable

## Abbreviations

NASH: Non-alcoholic steatohepatitis
NAFLD: Non-alcoholic fatty liver disease
CRF: Case report form
CRA: Clinical Research Associate
SCReN: Spanish Clinical Research Network
IDIVAL: Institut of Research Valdecilla
AEMPS: Spanish Agency of Medicines and Medical Devices
EC: Ethics committee
BS: Bariatric surgery
BE: Bariatric endoscopy
LSG: Laparoscopic sleeve gastrectomy
ESG: Endoscopic sleeve gastroplasty
